# Contingent reflections on coronavirus and priorities for educational planning and development

**DOI:** 10.1007/s11125-020-09480-3

**Published:** 2020-06-26

**Authors:** Keith M. Lewin

**Affiliations:** University of Sussex, Sussex House, Falmer, Brighton, BN1 9RH UK

**Keywords:** Development, Educational planning, Learning, Pandemic

## Abstract

The COVID-19 pandemic has dramatically shifted education and development priorities. The tragic death toll and high rates of morbidity across many countries are an unprecedented setback and a calamity for those affected physically and mentally. The economic and social effects of lockdowns, loss of production and business confidence, and global recession will cast a long shadow over education systems. Despite the 435 million items that Google already indexes under “COVID-19 education”, many things remain unknown. No one has a clear idea of how the current pandemic will unravel over anything but the short term. The challenge is to strengthen the mechanisms to separate evidence from opinion and to balance popularism with speaking truth to power—especially when political systems can find it difficult to distinguish fact from convenient fiction.

This paper advances ten propositions that will shape policy dialogue and whatever iteration of the Sustainable Development Goals is needed to ensure they remain fit for purpose. Testing these propositions over the next year will open the door to an evidence-based approach to reconstruction and sustainable development and juxtapose immediate concerns of the present with aspirations for the future. More than ever the need is to see beyond the exigencies of COVID-19 and act to secure the educational gains of the recent past. UNESCO and other development agencies can play a key role in sharing how to manage revitalised and resilient learning systems that allow the community of practice to stay focussed on development that is economically, socially, medically, and educationally sustainable. Spaceship Earth has to be secured for future generations through the knowledge, skill and attitudes of its crew and passengers. Seriously revisiting SDG4, its targets and indicators, and its relationships with other SDGs, would be a start.

The COVID-19 is reshaping the education and development debate. However, the simple truth is that no one has a clear idea of how the current pandemic will unravel over anything but the short term. Despite the 435 million items that Google already indexes under “COVID-19 education,” and a surprising 1.4 million hits for “COVID-19 Educational Planning”, many things remain unknown. These include whether (and for how long) infection and survival result in a level of immunity, the likelihood that a vaccine can be created and mobilised for mass immunisation, the probability of more effective therapies, and the possibility of subsequent waves of infection with or without mutations in the virus. The list of needs is long and there has been an avalanche of advocacy promoting different approaches to manage the short-term effects of COVID-19 on schools, children’s learning, teachers’ safety, and the mental health of all those involved in education. Less has been said about the long term effects of economic recession on educational financing though that may be the most chronic impact of the pandemic stretching long into the future.

Scientific data on the virus’s epidemiology, pathogenesis, and morbidity is critical to evidence-based policy on everything from the need for lockdowns, the appropriate use of Personal Protective Equipment (PPE), and prudent operating procedures for schools and other public institutions. Some of the understanding that is emerging will apply across all populations, with a high degree of universal applicability. Other understandings will have to be mediated by context, capacity, demography, resources, and the political economy of the possible.

Communities of practice in education have never been short of opinion leaders, influencers, and advocates. These include commentators with credible qualifications and experience to understand and predict how this unique pandemic will evolve, but also those who opportunistically use the events around COVID-19 to promote favoured ideologies, contentious narratives of the spread of infection, and predictions of different futures without a secure evidential base. The challenge is to strengthen the mechanisms to separate evidence from opinion and to balance popularism with speaking truth to power—especially when political systems can find it difficult to distinguish fact from convenient fiction.

There is an emerging consensus that children seem least affected by the COVID-19 while older adults are most at risk, especially those with co-morbidities and those suffering from social disadvantage. A recent University of Cambridge study (Lee 2020) indicates that the risk of children under 15 years old catching and dying of COVID-19 in the UK is over one in 5 million, about 75 times smaller than dying in a car crash. In contrast, the risk of infection and death for people over 90 years old is about 1 in 80. Rates of infection and morbidity appear highest in households with poor socio-economic indicators and amongst some social groups, but attributing causality is proving complex since occupational group, civic status, educational levels, ethnicity, and income all interact.

It is clear that the exponential growth of infection and subsequent morbidity and mortality have peaked in those countries which experienced the pandemic first, and new cases appear to be in decline. The numbers will change and there remains a risk of second and third waves of infections, but there are now many examples of countries which appear to have been able to manage the infection rate (the R ratio) downwards, indicating that the number of new cases every day is falling.

Optimists will plan on the basis that these trends will continue, whilst being vigilant of any regression back to exponential growth patterns. There can be no misunderstanding. The death toll and high rates of morbidity are an unprecedented disaster and a calamity for those affected physically and mentally. The economic and social effects of lockdowns, loss of production and business confidence, and global recession will cast a long shadow over development. COVID-19 is deeply disruptive and requires reconsideration of development priorities, managed pathways back from the suspension of school systems, and new investment in global public goods, including education systems.

Ten propositions can be advanced that can help shape policy dialogue for the post-COVID-19 future. Testing these propositions over the next year will open the door to an evidence-based approach to reconstruction and sustainable development, one that can juxtapose the immediate concerns of the present with aspirations for the future to build on the gains of the past.

## *Proposition 1: The basic arithmetic of organising and financing and mass education systems will remain structurally the same as it was before COVID-19.*

Mass provision of education will continue to be provided through schools that are publicly funded, especially for the poor and marginalised. The public good aspects of extending learning opportunities to all will remain undiminished, though it will need to be closely coupled to reducing the inequalities that COVID-19 will exacerbate. These inequalities include asymmetry in the loss of opportunities to attend school, extreme variations in the capacity of households to homeschool children, and uneven disruptions to teacher deployment and absenteeism, especially where risks to teachers are real and unrecognised. It will remain true that Low and Low Middle Income countries (LICs and LMICs) will need to spend 6% of GDP and to collect more than 25% of GDP in revenue to achieve and sustain and universal enrolment in schools of minimal quality (but see Proposition 10 below).

## *Proposition 2: The amount of grant aid from donors will plateau and may fall, as the appetite for aid to education comes under increasing pressure from new domestic priorities and economic recession.*

The domestic political economy of development assistance in donor countries will diminish willingness to give grant aid, accelerating existing trends (Lewin 2019). Investments in health care systems and global health will take precedence over access to education and learning. Tied aid will become more common as rich countries seek to rebuild their own employment and corporate revenues. Aid sponsorship of higher education students to attend universities in the OECD in person will suffer from restrictions on travel. In the short term, domestic tax revenue will fall in OECD countries as the effects of economic lockdowns and global recession work their way through. This will reduce commitments to aid for education in real terms as government budgets and GDP shrink. Fiscal reforms that can increase domestic revenue, especially where revenue is low, e.g. in LICs and LMICs, will become even more pressing. Though grants related to immediate needs created by the pandemic can be useful (GPE 2020), they cannot meet needs for recurrent financing. Nor is it clear if it would be better to direct such grants to address short term lack of access to conventional schools, or towards more intensive care beds and ensuring that test, track and trace is effective in controlling the spread of new infection.

## *Proposition 3: LICs and LMICs will suffer more than OECD countries from reductions in domestic revenue, making educational financing more difficult.*

Domestic economic activity is being compromised by necessary lockdowns, morbidity has a direct effect on productivity, falls in demand for exports are reducing taxable transactions, and income and tourist revenues are collapsing. Consequently, the collection of personal income tax will fall as employment shrinks, especially where PAYE systems are weak. VAT receipts will drop as consumption falls. Corporate taxes will follow the downward economic cycle. Remittances will drop dramatically, with a huge impact on LICs and LMICs that export labour. In most LICs and LMICs savings will not be sufficient to buffer recession. The temptation is to borrow to finance recurrent public expenditures, as has happened in the OECD. But the risks for sub-prime borrowers in LICs and LMICs, with already high debt-to-GDP ratios and poor credit ratings, are much greater. Loans and development bonds can have dangerously high interest rates of 8% or more annually before transaction costs. This can result in more interest being paid than the value of the original loans. This benefits the lender more than the borrower. As Ibsen observed in *A Doll’s House*, “There can be no freedom or beauty about a home life that depends on borrowing and debt” (Ibsen 1992).

The experience of debt relief under the Heavily Indebted Poor Countries (HIPC) initiative since 1996 is illustrative of the risks of over borrowing. As recently as the end of March 2020 the International Monetary Fund warned that “to reverse the recent increase in LIC public debt burdens and reduce their debt vulnerabilities, countries need to pursue cautious borrowing policies and strengthen their public debt management” (IMF 2020). Debt write off linked to mitigation of COVID-19 impact is widely mooted. This makes good sense but only if egregious lenders are not protected from the consequences of their previous actions by the translation of private to public debt that is then written off. This creates moral hazard and an incentive for future sub-prime lending.

## *Proposition 4: Education systems will reopen and will rebuild themselves in ways that largely replicates their existing form, with more attention to resilience in the face of systemic risk.*

 Assuming COVID-19 is mitigated and morbidity falls to levels similar to the aftermath of previous pandemics, the most likely outcome in five years’ time is that the existing structures of mass education systems will adapt and persist. Organisational forms, physical structures, working practices, and roles in social reproduction and economic development are deeply embedded and widely thought to be functional. Despite the many critiques of the characteristics of education systems and regular attempts at radical reform, schools, colleges, and universities persist in forms that converge more than they diverge. Evolution is more likely than revolution in system development (Lewin 2015a). The most advanced economies have yet to replace schools with other forms of educational process and their elite institutions celebrate conventional forms of education and training. Most LICs and LMICs seek to replicate the dominant forms of schooling in high-income countries and imitate forms originating in OECD countries.

Notwithstanding this overall observation, there will be changes. Social distancing will become common, teachers may need Personal Protective Equipment (PPE) in some circumstances, distance learning technologies will emerge where they can operate consistently at affordable costs, and assessment may make more use of virtual than real testing environments with less practical work. The opportunities to innovate should be grasped, but so also should lessons be learned about how to invest in making education systems more failsafe. More “just in case” measures may need to replace their “just in time” counterparts. Planning will need to place more emphasis on resilience, developing robust and distributed systems of learning that are less centralised and dominated by narrowly defined cost efficiency and high-stakes testing.

## *Proposition 5: The global pandemic has illustrated the importance of global public goods like health and education.*

Almost all countries have had public health campaigns to manage COVID-19. These require skilled staff in public health systems that generate and validate health messages, as well as a literate and numerate population capable of understanding and acting on such messages. The crisis has highlighted how public institutions are at the heart of a response to the crisis that requires collective action, not to mention responsible sharing of the burden of disruption, the risks of infection, and ultimately treatment. Without public goods that are publicly financed, hospitals cannot reach beyond those who can pay for medical services, and all children cannot access educational services that are rationed by price. Key workers need to continue to send their children to school if they are to continue to work. More efficient and effective use of trained teachers, secure in their environments, will be an overdue benefit.

## *Proposition 6: Non-state providers have to develop business plans that factor in a new economics of private school operation.*

Private schools depending on fee-paying students will have to manage a significant loss in income as disposable household income is squeezed. Austerity will have a disproportionate effect on middle- and lower-income families, where effective demand for fee-paying private schooling can be expected to fall as incomes are reduced. It may also be reflected in gendered preferences to enrol and other social exclusions of relatively powerless groups. New arrangements for social distancing in classrooms, PPE for teachers and pupils, boarding, transport, and sanitation and hygiene are likely to inflate costs. Private providers may seek subsidies from states to maintain viable businesses during and beyond lockdown, but most LICs and LMICs will not be able to finance salary supplements and loan repayment holidays. It will be a political question as to whether the opportunity costs of diverting public resources for this purpose are judged to be socially beneficial.

## *Proposition 7: Children currently not attending school as a result of school closures by governments should not be confused with conventionally defined Out of School Children (OOSC).*

Their causalities are unrelated, and the numbers affected are vastly different. A precipitate rush into distance education, artificial intelligence driven internet schooling, or one of many versions of homeschooling is both unrealistic and unwise, at least until the temporary and permanent effects of COVID-19 can be distinguished from each other. It will be a mistake to adopt new priorities in a hurry with only opinion as a foundation for action. Existing priorities remain important. Conventionally defined OOSC excluded by the cost of education, location, civic status, or disability continue to be at risk. Learners not learning before the crisis still have the same need to learn.

## *Proposition 8: Teachers have to be seen as key workers, and schools as key institutions.*

Teachers have not been considered “key workers” in most countries, except to meet the need to keep some schools open for the children of other key personnel so that they can go to work. In the short term, the consensus in most countries is that closing schools made good sense. Before it was known that children were at low risk of severe illness, the precautionary principle dictated that public authorities should err on the side of caution. Schools are key social institutions that create meeting spaces for teachers and parents who would clearly be at greater risk than if they were self-isolating. As infection rates fall, schools will continue to play a central role in the ability of societies to reproduce themselves, transmit knowledge and skill across the generations, and encourage behaviour based on evidence rather than superstition and commercial interest. Teachers as key workers have to take the lead on developing safe practices that reflect the evidence and that recognise the need for consensus about acceptable levels of risk. Schools, as key public institutions, can demonstrate how COVID-19 can be managed in institutions, building on their experience with more familiar infection and morbidity risks.

## *Proposition 9: Sustainable Development Goal 4 (SDG4) for education needs to be revisited in the light of the dislocations of COVID-19.*

The world is now seriously off track to achieve SDG4 (UN 2015). The *force majeure* of the pandemic is causing dislocations. There are many consequences for the achievement of this Goal. These include: i) children de-schooled by temporary closures may not return, especially if they are already at risk of drop out; ii) ambitions to extend universal schooling to grade 12 look premature, given the direct and indirect costs and likely falls in levels of employment and household incomes; iii) demand for technical and vocational education will fall as labour markets contract; iv) equitable opportunities to learn are threatened by instabilities in school income, staffing, and safety; v) universal literacy may have less utility as modern sector development slows; and vi) global citizenship is challenged by closing borders and restrictions on movement of workers and students. Simply changing the SDG time frame to allow a longer period to achieve the same goal and targets is unlikely to be the best strategy, but it is the most probable. After five years of striving to achieve SDG4, a comprehensive review is needed. This must revisit whether the Goal and its ten targets are still fit for purpose, whether some targets should be deprioritised in the light of recent events and others introduced, and how the Goal and targets should be adapted now to the radically different country circumstances created by COVID-19.

## *Proposition 10: The SDG4 indicators also need to be changed, even if SDG4 and its Targets are retained.*

Useful targets need to be achievable and target setters must co-commit with target getters to common indicators that are relevant to different systems. This now includes indicators that have some bearing on COVID-19 status. What is important to achieve is dependent on where a country and education system is located on the pandemic curve. Thus, measuring changes in enrolment rates during a system closure seems pointless. Learning levels need assessing in a different way before and after disrupted schooling. Gender equity may look different as a result of changes in social care. Other indicators of progress need careful reconceptualization. For example, one of the SDG4 ambitions is that LICs and LMICs will allocate around 6% of GDP to education (from a current average of around 4%) and that the proportion of the public budget that governments spend will increase to 20% (from an average of about 15%). These changes will almost certainly happen in many countries in 2021. But it will not mean more money spent on education. This is because GDP in many LICs and LMICs will fall and at the same time educational spending will prove sticky on the downturn. Education system costs are mostly in teachers’ salaries and these will continue to be paid. The exception will be in systems which have casualised the teaching profession with short-term contract teachers, and where public and private providers employ teachers on a temporary basis with few contractual obligations. In these cases, resilience will be low. The SDG4 indicator that is the percentage of GDP spent on education is thus misleading. It can increase in value when less money is spent on education if GDP itself shrinks. It needs replacing.

The pandemic is nowhere near over. It will not be resolved by the time this article is published. COVID-19 may return in a more or less virulent form. History suggests that all pandemics dissipate, and that underlying trends of development reappear with a degree of continuity with the past (Shaw-Taylor 2020). Whatever is said at this point in time has to be mediated by evidence of changing circumstances as it emerges. Global prescriptions have to be matched by local interpretations of what works best in context. Education systems have a special responsibility to disseminate and interrogate evidence on COVID-19, so that real facts are not overshadowed by superstition, self-interested bias, and electoral expedience.

Until it becomes much clearer what aspects of social and economic organisation really have irreversibly changed, which new developments are functional and durable, and which have the support of effective demand rather than the fragility of supply-pushed solutions, the smart money is on incremental recovery and development strategies closely coupled to feedback on effects. Radical departures that imagine away inconvenient truths about the difficulties of educating *all* the children, overlook growing inequalities, and put faith in simple solutions to complex problems risk undermining much of what has been achieved over the last three development decades. UNESCO and other multi-lateral agencies can play a key role in sharing how to manage revitalised learning systems that allow us to stay focussed on development that is economically, socially, medically, and educationally sustainable, and that preserve Spaceship Earth for future generations (Lewin 2015b). Seriously revisiting SDG4 and its targets and indicators, and its relationships with other SDGs and economic recovery from global recession, would be a start.
